# Full Glowworm Swarm Optimization Algorithm for Whole-Set Orders Scheduling in Single Machine

**DOI:** 10.1155/2013/652061

**Published:** 2013-10-31

**Authors:** Zhang Yu, Xiaomei Yang

**Affiliations:** ^1^Computer Science and Technology School, Taiyuan University of Science & Technology, Taiyuan 030024, China; ^2^Economics and Management School, Taiyuan University of Science & Technology, Taiyuan 030024, China

## Abstract

By analyzing the characteristics of whole-set orders problem and combining the theory of glowworm swarm optimization, a new glowworm swarm optimization algorithm for scheduling is proposed. A new hybrid-encoding schema combining with two-dimensional encoding and random-key encoding is given. In order to enhance the capability of optimal searching and speed up the convergence rate, the dynamical changed step strategy is integrated into this algorithm. Furthermore, experimental results prove its feasibility and efficiency.

## 1. Introduction

Whole-set orders problem refers to customers' orders including multiple workpieces with different processing time and completion deadlines; since these workpieces are matching together as one, the delivery delay of the whole order will account for one delayed piece; customers are meeting a matching problem. In the customized production environment, whole-set orders problem can better reflect the corporations' service level and customers' satisfaction. It has become an important branch in the field of production scheduling and has broadened practical backgrounds.

At present, the algorithms used in production scheduling can be divided into accurate algorithms and approximation algorithms. Accurate algorithms (mathematical programming, branch and bound algorithm, Lagrangian relaxation, etc.) can get accurate solutions of problems, but big amount of calculation and time-consumption limit their applications in solving small-scale problems. Approximation algorithms (genetic algorithm, particle swarm optimization, ant colony algorithm, etc.) for their simple operation, parallel processing, have been widely applied in production scheduling and large-scale problems.

Glowworm swarm optimization (GSO)  is proposed by Krishnanand and Ghose as one of the newest nature inspired heuristics [1], with it's simple model, less adjustable parameters, and fast convergence rate, which can be usually viewed in pattern recognition, routing, combinatorial optimization, and so forth [2–6]. In the optimizations of production scheduling, Kazem Sayadi et al. have successfully gotten the better solutions of permutation flow-shop scheduling problem [7]. Wu et al. have proved its feasibility and efficiency in the optimization of cross-dock scheduling [8]. Based on the discrete characteristic of whole-set orders and GSO's good performance in discretization, this paper presents an improved GSO for whole-set orders scheduling problem.

## 2. The Description of Weighted Whole-Set Orders Problem

### 2.1. The Model of Whole-Set Orders Problem in Single Machine

Due to the definition of whole-set orders problem, when each order includes only one workpiece, it becomes a problem of weighed number of delay jobs, so whole-set orders problem is a kind of NP-hard problem. 

Maximizing the number of weighted whole-set orders is our objective function:

(1) $$\begin{array}{c} f=\max\sum_{h=1}^{H}w_{h}\;x_{h}. \end{array}$$

We suppose the following.

1. There are *N* independent workpieces that need to be processed on one machine and belong to *H* orders, *G*_1_, *G*_2_,…, *G*_*H*_, *G*_*h*_ (1 ≤ *h* ≤ *H*) includes *j*_1_^*h*^, *j*_2_^*h*^,…, *j*_*n*_*h*__^*h*^ jobs, ∑_*h*=1_^*H*^ *n*_*h*_ = *N*, the weighted of *h* is *w*_*h*_.

Consider

(2) $$\begin{array}{c} \sum_{h=1}^{H}w_{h}=1. \end{array}$$

(1) The processing time of workpiece *j*_*j*_^*h*^ (1 ≤ *h* ≤ *H*, 1 ≤ *j* ≤ *n*_*h*_) is *p*_*j*_^*h*^ (>0), and deadline is *d*_*j*_^*h*^.
(2) All the workpieces are got ready, which means arriving time *r*_*j*_^*h*^ = 0.
(3) One piece can only be processed once, and single machine  should process one job each time, *a*_*ij*_ stands for if job *i* was processed at the position of *j*, then *a*_*ij*_ = 1, else
  (3) $$\begin{array}{c} a_{ij}=0\;,\;\sum_{n=1}^{N}a_{ij}=1,\;j=1,2,\ldots ,N. \end{array}$$
(4) Once processed the workpiece should not be terminated.
(5) The complete time of workpiece *j*_*j*_^*h*^ is *C*_*j*_^*h*^, and it defines *x*_*h*_ for whole-set coefficient as
  (4) $$\begin{array}{c} x_{h}=\{\begin{array}{cc} 1, & \text{if}\;\sum_{j=1}^{n_{h}}U_{j}^{h}=n_{h},\\ 0, & \text{else}, \end{array}\;\;U_{j}^{h}=\{\begin{array}{cc} 1, & \text{if}\;\;C_{j}^{h}\leq d_{j}^{h},\\ 0, & \text{else}. \end{array} \end{array}$$

### 2.2. Characteristics of Whole-Set Orders Problem

The characteristics of whole orders problem include its complexity, restriction, and discreteness.

(1)  *Complexity*. For a machining sort with *n* workpieces, there may be *N* factorial solutions. For example, if we get 7 customers, and 20 workpieces to machining, the total number of the solutions will be 2.4329*e* + 18. This reflects that with the enlargement of the scheduling scale, the space of solutions will become lager, and the computation will increase exponentially. This needs to keep the diversity of metapopulation in solving whole-set orders problem, to shorten the solving time, to increase the probability of acquiring optimal solution and, to realize global optimization.

(2)  *Restraintion*. As the optimal solution must meet the machine's or processing sequences' restraint conditions in whole-set orders problem, part of the sorts may become unfeasible scheduling solutions for not meeting the restraints. We should note metapopulation individual's validity in searching process when using glowworm swarm optimization and adopt revise strategies to unfeasible individual coming from location update to ensure the feasibility of the descendant. 

(3)  *Discreteness*. In classical GSO, the mobile step is usually a fixed numerical value. And this has good effect on solving continuous optimizing problems. But every metapopulation individual represents an independent panel point in whole-set orders problem, and unreasonable setting of the step may lead to mismatching situations in searching process. So in order to ensure the convergence effectiveness, we should do some dynamic handlings on step.

## 3. Glowworm Swarm Optimization for Whole-Set Orders Scheduling

### 3.1. Description of Classical Glowworm Swarm Optimization

Most kinds of glowworms can locate its position and exchange information by sending out rhythmed short beam. The idea of GSO is glowworm individual finding flaring neighbors in its searching scope. Move from initial position to a better one and at last assemble into one or more extreme value point.

In GSO algorithm, Glowworm individuals' attraction is only related to its brightness. Attraction of individual is proportional to brightness and inversely proportional to the distance between the two individuals. The position of individuals account for objective function value. Define dynamic decision domain as individual searching scope. When updating position, individuals move by step. 

Detailed procedures of classical GSO are as follows.

(1)  Initialize parameters. *n* individuals are randomly placed in feasible region, *l*_0_ accounts for fluorescein value, *r*_0_ for dynamic decision domain, *s* for step, *n*_*t*_ for threshold in domain, *ρ* for fluorescein elimination coefficient, *γ* for fluorescein update coefficient, *β* for update coefficient of domain, *r*_*s*_ for maximal searching radius, and *t* for iteration number.
(2)  The objective function value *J*(*x*_*i*_(*t*)) is transformed to *l*_*i*_(*t*) as
  (5) $$\begin{array}{c} l_{i}\left(t\right)=\left(1-\rho \right)l_{i}\left(t-1\right)+\gamma J\left(x_{i}\left(t\right)\right), \end{array}$$
 in which *x*_*i*_(*t*) accounts for the position of individual *i* at *t* time.
(3)  In each *r*_*d*_^*i*^(*t*), select higher fluorescein value individuals forming a set of neighborhood *N*_*i*_(*t*). Hence,
  (6) $$\begin{array}{c} N_{i}\left(t\right)=\left\{j:||x_{j}\left(t\right)-x_{i}\left(t\right)||\leq r_{d}^{i}\left(t\right);l_{i}\left(t\right)\leq l_{j}\left(t\right)\right\}. \end{array}$$
(4)  The probability of individual *i* may move toward *j* as
  (7) $$\begin{array}{c} p_{ij}\left(t\right)=\frac{l_{j}\left(t\right)-l_{i}\left(t\right)}{\sum_{k\in N_{i}(t)}l_{k}\left(t\right)-l_{i}\left(t\right)}, \end{array}$$
 in which *j* is chosen by *p*_*ij*_(*t*).
(5)  The position of individual *i* can be updated as
  (8) $$\begin{array}{c} x_{i}\left(t+1\right)=x_{i}\left(t\right)+s\left(\frac{x_{j}\left(t\right)-x_{i}\left(t\right)}{||x_{j}\left(t\right)-x_{i}\left(t\right)||}\right). \end{array}$$
(6)  The dynamic decision domain can be updated as
  (9) $$\begin{array}{c} r_{d}^{i}\left(t+1\right)=\min\left\{r_{s},\max\left\{0,r_{d}^{i}\left(t\right),\beta \left(n_{t}-\left|N_{i}\left(t\right)\right|\right)\right\}\right\}. \end{array}$$

### 3.2. Glowworm Swarm Optimization for whole-set orders Scheduling (GSOS)

By analyzing the characteristics of whole-set orders problem, this paper will solve it by using glowworm swarm optimization. The key of this algorithm includes encoding and decoding schema, individuals standing without neighbors, variable step strategy, and the distance of individuals.

#### 3.2.1. Encoding and Decoding Schema

A sound encoding method can lower solving difficulty caused by constraint conditions and raise efficiency. The common encoding methods in approximation algorithms at present include machine-based coding and process-based coding. Among these methods, machine-based coding can reflect limitations of the processing machines, while it may easily result in deadlock scheduling; Jobs' relation-based coding uses binary coding method which may easily cause redundant scheduling results. Process-based coding assigns the same symbol to every process of the same job, but the result producing through this method is not an initiative scheduling.

In view of this, a hybrid encoding schema combining with 2-dimensional coding and random-key coding is given. In whole-set orders problem, one individual's location accounts for one feasible code, and the movement of individual means the exchange of codes.

In the coding, the method in this paper combines natural numbers and randomly real numbers between 0 and 1. Natural numbers 1,2,3,…*n* stand for *n* piece, and  *x*_*i*_ stands for positive real numbers between 0 and 1 without repetition generated randomly. As is shown in Table 1.

This method increases bits of valid number. It could avoid some repetitive sorts that may lead to invalid codes. In decoding, relative size of number accounts for the process position of workpiece *i* in the sorts, ascending sort column (*x*_1_, *x*_2_, ..., *x*_*n*_), and the corresponding sort of natural numbers is the process sequence.

 In example 1, there are 6 workpieces and their 2-dimensional codes like Table 2.

There are 6 workpieces and their 2-dimensional codes like Table 2.

Ascending sort the real numbers we get 0.01-0.23-0.44-0.48-0.76-0.79; the code of workpiece number 1 is 0.23, and 0.23 is the second minimum in the sort, so workpiece 1 will be processed at second position. Successively analyzing, we can get the process sequence: 4-1-5-2-3-6.

Furthermore, all the feasible codes and process sequences are one-to-one correspondence. Because of the difference of each real number, their ascending sort would be uniqueness.

#### 3.2.2. Dispose of Individual without Neighbors

According to classical GSO, an individual glowworm performs random move towards a neighbor better than itself through probability in accordance with the neighborhoods' fluorescent brightness. If there are too many candidate solution or the neighborhoods distribute unevenly, there may be a small probability of the event that some individuals do not have neighbors so that they would like to be stagnant, which may results in the slowing down of the rate of convergence and the likely access into the local optimization. To avoid the above disadvantages, every individual must be ensured to be dynamic in the optimizing process. Thus, in this algorithm, if an individual has no neighbor, it should move one step at random in the optimizing process of its own generation.

#### 3.2.3. Strategy of Improved Moving Step

The step size of an individual in GSO is ordinarily fixed. In solving the whole-set orders problem or similar special problems, both bigger and smaller steps will cause adverse effects in that the bigger steps may result in missing the optimal solution, slowing down of the rate of convergence, and the easy occurrence of shaking, while the smaller steps may result in an early trapping in the local optimization by which the optimal solution cannot be obtained. Therefore, changing the step size dynamically can improve the solving efficiency of the algorithm.

**Figure:**
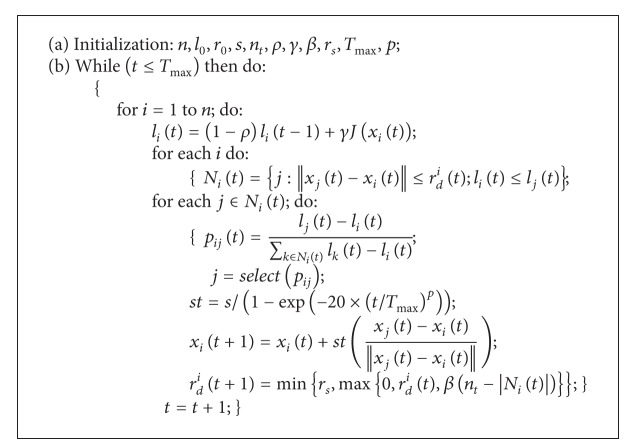
PSEUDOCODE 1

**Figure 1:**
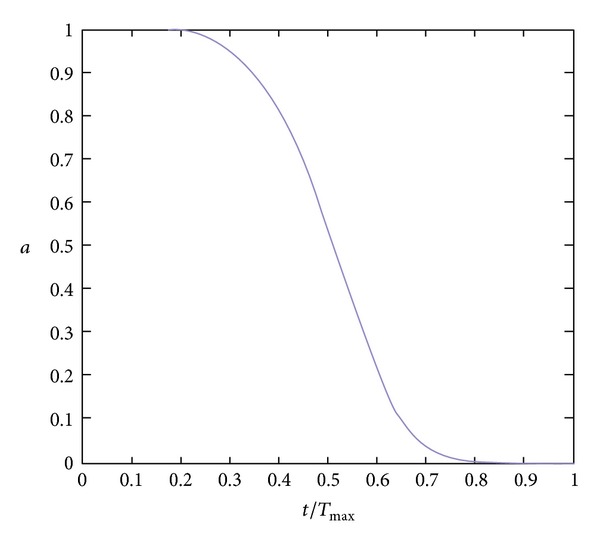
Curve of *a*.

At the beginning of the algorithm, the step size should be kept big enough to avoid trapping into the local optimization. With the increasing of iteration, the step size should be shortened gradually to ensure an optimal solution at the later stage of the algorithm. So the formula of moving step is designed as

(10) $$\begin{array}{c} \left(1-a\right)*st=s,\\ a=\exp\left(-20\times {\left(\frac{t}{T_{\max}}\right)}^{p}\right), \end{array}$$

in which *s* accounts for minimum step and can be initialized, *T*_max⁡_ for maximum iteration, *t* for current iteration, *p* and for a integer between [1, 30]. Let *p* = 5; the curve of *a* is shown as Figure 1.

#### 3.2.4. Formula of Distance between Individuals

In whole-set orders problem, bit of codes is related to the numbers of workpiece and usually may be multidimensional array, and the key of it is how to calculate distance between individuals. Here, we suppose there are *N* workpieces to be processed, and finally get two feasible sequences after decoding as follows:

(11) $$\begin{array}{c} x_{i}=\left(x_{i1},x_{i2},...,x_{iN}\right),{\;\;x}_{j}=\left(x_{j1},x_{j2},...,x_{jN}\right), \end{array}$$

the distance from the symmetry of formula is obtained as

(12) $$\begin{array}{c} d_{ij}=\frac{\left|x_{i1}-x_{j1}\right|c+\left|x_{i2}-x_{j2}\right|c+\cdots +\left|x_{iN}-x_{jN}\right|c}{N\left(N+1\right)/2}, \end{array}$$

in which *d*_*ij*_ = *d*_*ji*_. Thus, *c* is usually fixed, and we make it to be 4 in the experiments.

For example, there are two process sequences after decoding (6-5-4-3-2-1) and (1-2-4-3-6-5). By *d*_12_ = 4∗{(|6 − 1 | +|5 − 2 | +|4 − 4 | +|3 − 3 | +|2 − 6 | +|1 − 5|)/(6∗(6 + 1)/2)} = 64/21, the distance needed is obtained.

The pseudo codes of GSOS as shown in Pseudocode 1.

## 4. Simulations and Results

To verify the feasibility of GSOS, we have tested two simulations of different scales.

**Case 1**

According to [9], there are 41 workpieces of 10 orders, detailed information are shown in Tables 3 and 4; target of optimization is maximizing the number of weighted whole-set orders.

In Table 4, numbers in row-1 account for deadlines, in line-1 account for orders and in brackets for processing time. According to the above information, we contrast the results between GSOS and GA. Simulation environment: Microsoft Windows-XP system, AMD-A6-3400M CPU, 2G-RAM, and the codes are programmed by MATLAB2012a, the population size is fixed *n* = 400, maximum iteration *T*_max⁡_ = 80, and the program run 10 times independently.

The efficiency of results is shown in Table 5. From Table 5, we can find that average solving time of GSOS is shortened by about 34.74 seconds compared with GA, which increases by about 29.3%.

The results of optimization numbers are shown in Table 6. Form Table 6, we realize that GSOS performs better than GA in terms of average value, minimum value, and variance.

**Case 2**

To insure the performance of GSOS, Example 2 is generated randomly, information is in detail in Tables 7 and 8.

The results of optimization numbers are shown in Table 9, and the efficiency of results are shown in Table 10.

From Tables 9 and 10, we can find that the proposed algorithm is better than GA.

**Figure 2:**
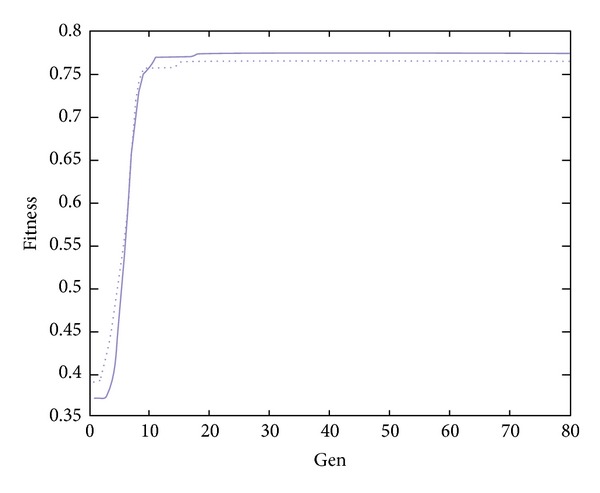
Curves in case 1.

**Figure 3:**
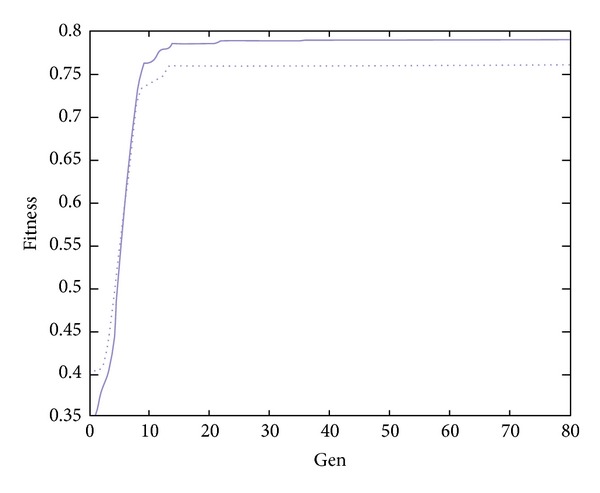
Curves in case 2.

The searching curves of GSOS and GA are shown in Figures 2 and 3, there solid lines account for GSOS while dotted lines for GA.

According to the Figures 2 and 3, GSOS and GA both have a high rate of convergence, but GSOS performs better than GA in terms of average value, minimum value, and variance, which proved its high-accuracy of solutions, the solving time of GSOS decreases by about 29% compared with GA which reflects its efficiency. In conclusion, GSOS is more suitable for solving whole-set orders problem.

## 5. Conclusions

An improved glowworm swarm optimization for scheduling (GSOS) is proposed in this paper; we have verified its high rate of convergence, efficiency, accuracy, and easy operation through simulations on different scales of whole-set orders problem. To test its performance on parallel machines and bigger scales that will be our research direction later on.

## Figures and Tables

**Table 1:** 2-dimensional individual encoding.

1	2	3	⋯	⋯	*n*

*x*_1_	*x*_2_	*x*_3_	⋯	⋯	*x*_*n*_

**Table 2:** Case of codes.

1	2	3	4	5	6

0.23	0.48	0.76	0.01	0.44	0.79

**Table 3:** Orders and weights.

Order	1	2	3	4	5	6	7	8	9	10
Weight	0.08	0.1	0.09	0.11	0.12	0.2	0.05	0.15	0.05	0.05

**Table 4:** Information of orders.

	2	3	7	10	14	16	19	30	38	42	52	60
1	(1)		(2)			(2)		(2)		(2)		(2)
2	(1)			(2)	(1)						(2)	
3		(1)					(2)		(2)			(2)
4		(1)	(2)		(2)			(1)			(2)	
5		(1)				(1)				(2)		(2)
6				(1)			(2)					(2)
7				(1)		(2)			(2)			
8				(2)	(1)			(2)		(1)	(2)	
9					(1)		(1)		(2)		(1)	
10					(2)			(1)				(2)

**Table 5:** Comparison of time consumption.

Algorithm	1	2	3	4	5	6	7	8	9	10	Ave
GA	121.8	117.9	118.2	117.6	118.2	117.5	119.6	117.9	117.8	118.6	118.5
GSO	84.90	83.70	83.81	83.15	83.39	83.72	84.19	83.63	83.54	83.52	83.76

**Table 6:** Results of numbers.

Algorithm	Max	Min	Ave	Var
GA	0.77	0.68	0.727	0.0762
GSOS	0.77	0.72	0.752	0.0531

**Table 7:** Orders and weights.

Order	1	2	3	4	5	6	7	8
Weight	0.14	0.15	0.15	0.13	0.12	0.12	0.09	0.1

**Table:** (a)

Job	1	2	3	4	5	6	7	8	9	10
Order	1	1	1	2	2	2	3	3	3	4
Deadline	8	23	40	10	26	42	15	32	48	17
Cpu time	4.5	4	3.5	4	2	4	3	3	3.5	3

**Table:** (b)

Job	11	12	13	14	15	16	17	18	19	20	21
Order	4	4	5	5	5	6	6	7	7	8	8
Deadline	36	56	20	39	60	12	35	16	40	22	50
Cpu time	3	5	3.5	4.5	3	2	4	4	4	3	2

**Table 9:** Results of numbers.

Algorithm	Max	Ave	Var
GA	0.77	0.728	0.0493
GSOS	0.78	0.749	0.0491

**Table 10:** Comparison of time consumption.

Algorithm	1	2	3	4	5	6	7	8	9	10	Ave
GA	121.8	117.9	118.2	117.6	118.2	117.5	119.6	117.9	117.8	118.6	118.5
GSO	84.90	83.70	83.81	83.15	83.39	83.72	84.19	83.63	83.54	83.52	83.76
